# Supplementary figures and images for: Olig2 and Hes regulatory dynamics during motor neuron differentiation revealed by single cell transcriptomics
Source: PLoS Biol. 2018 Feb 1;16(2):e2003127. doi: 10.1371/journal.pbio.2003127 (PMC5811045; doi:10.1371/journal.pbio.2003127)

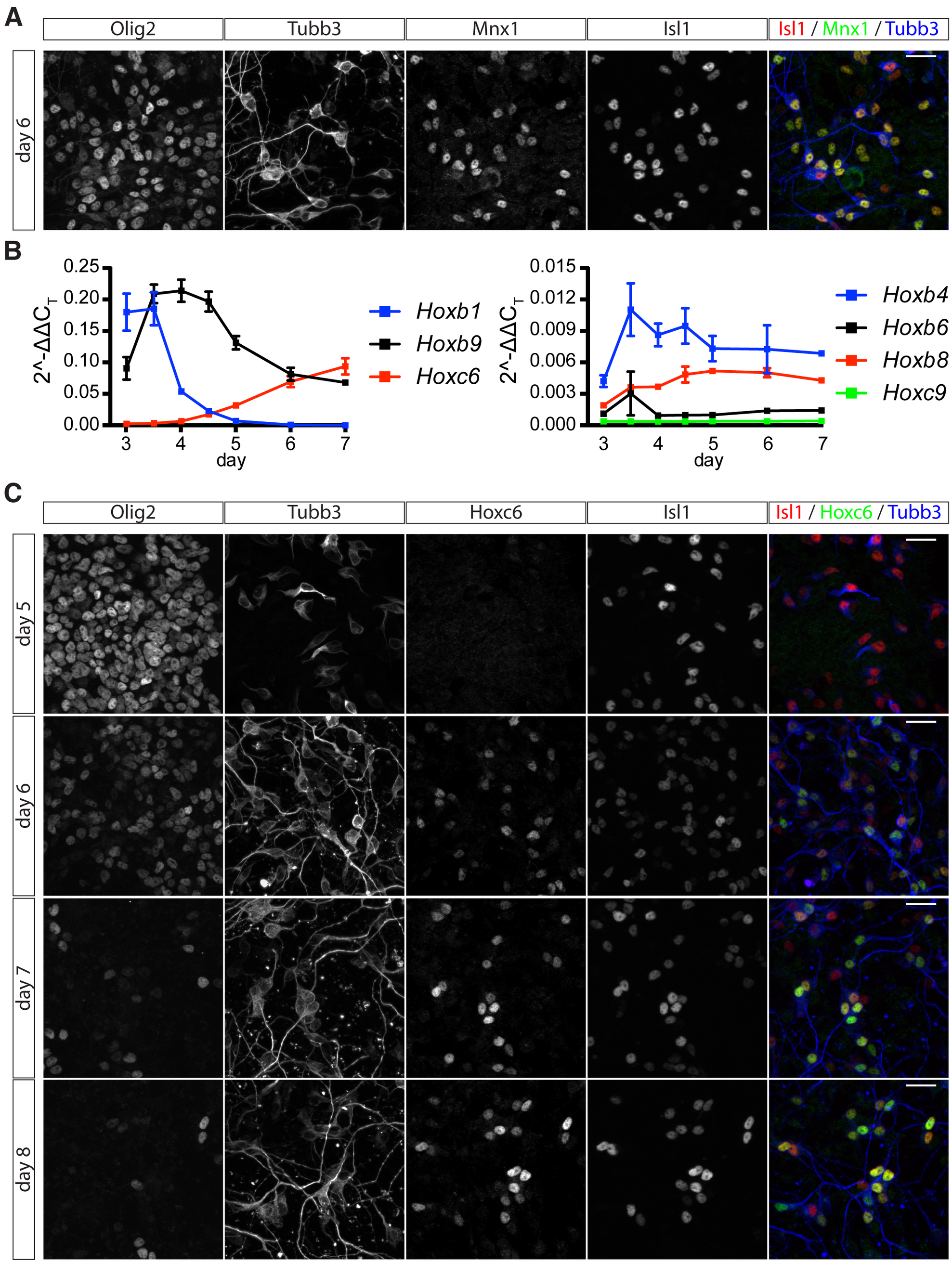

Supplement: S1 Fig — (A) Expression of the somatic MN marker Mnx1 in MNs at day 6. (B) RT-qPCR analysis of Hox genes expression levels from day 3 to day 7. Underlying data are provided in S1 Data. (C) Hoxc6 expression in MNs characterized by Isl1 and Tubb3 expression from day 6 to day 8. Scale bars = 40 μm. MN, motor neuron; NP, neural progenitor; RT-qPCR, real time-quantitative polymerase chain reaction; Tubb3, neuronal class III beta-tubulin. (TIF) [file pbio.2003127.s001.tif]

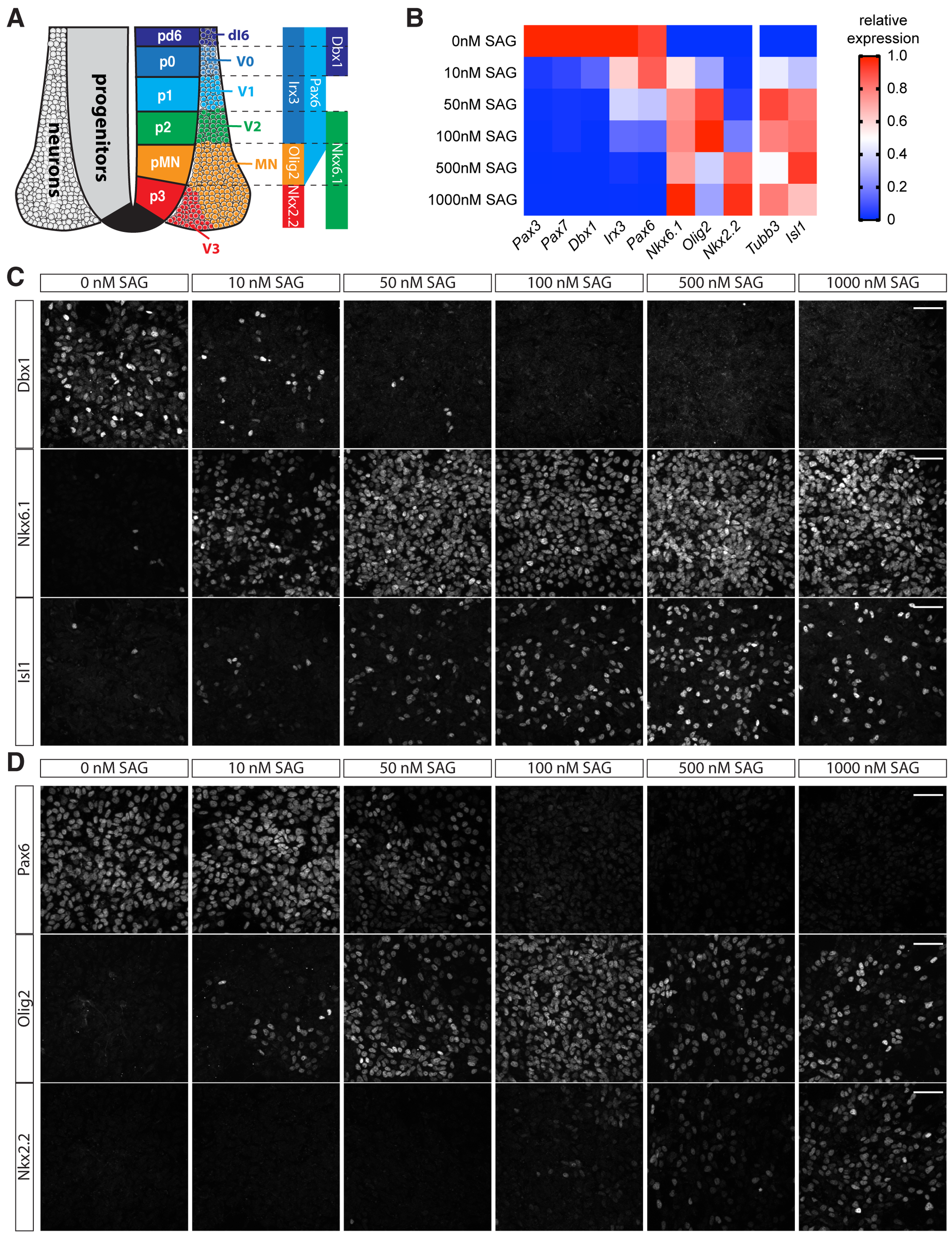

Supplement: S2 Fig — (A) Schematic of the embryonic spinal cord. Expression domains of TFs defining NP domains are indicated. (B) RT-qPCR analysis of day 6 differentiations treated with 0–1,000 nM SAG after day 3. (C) Expression of Dbx1, Nkx6.1, and Isl1 in day 6 differentiations treated with the indicated concentrations of SAG. (D) Expression of Pax6, Olig2, and Nkx2.2 in day 6 differentiations treated with the indicated concentrations of SAG. Scale bars = 40 μm. NP, neural progenitor; Shh, sonic hedgehog; TF, transcription factor; RT-qPCR, real time-quantitative polymerase chain reaction; SAG, Smoothened/Shh signalling agonist. (TIF) [file pbio.2003127.s002.tif]

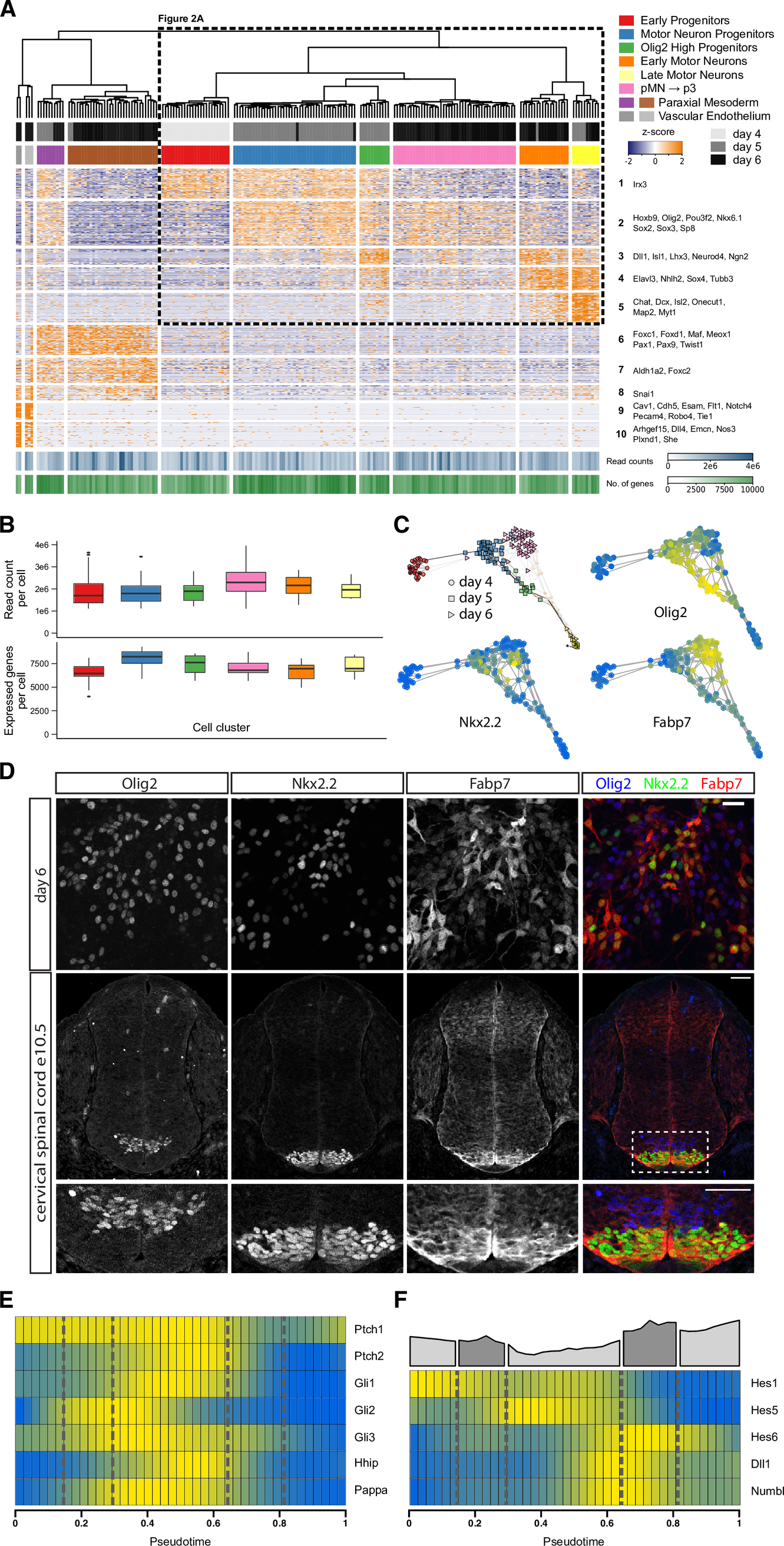

Supplement: S3 Fig — (A) Identification of cell states by hierarchical clustering from 202 cells based on 10 identified gene modules. Genes characteristic for the individual modules are indicated. The boxed region corresponds to the heat map in Fig 2A. (B) Quantifications of read counts per cell (top) and number of expressed genes per cell (bottom) for neural cell states identified by hierarchical clustering. Colors of the graphs match cell states in Fig 2A and S3A Fig. (C) Cell state graphs color coded for the expression levels of Olig2, Nkx2.2, and Fabp7. (D) Analysis of Olig2, Nkx2.2, and Fabp7 expression in differentiations at day 6 (top row) and e10.5 embryonic spinal cords (bottom row) confirms higher Fabp7 expression levels in p3 progenitors. (E) The transition phase from early Irx3 NPs to Olig2 NPs correlates with the induction of Shh target genes Ptch2, Gli1, Hhip. (F) Inhibition of Notch signalling, revealed by decreasing expression levels of Hes1/5 and expression of the Notch ligand Dll1 and the pathway inhibitors Hes6 and Numbl, identifies the cell state transition from NPs to MNs. Scale bars = 25 μm (D, top row) and 50 μm (D, bottom row). e, embryonic day; NP, neural progenitor; Shh, sonic hedgehog. (TIF) [file pbio.2003127.s003.tif]

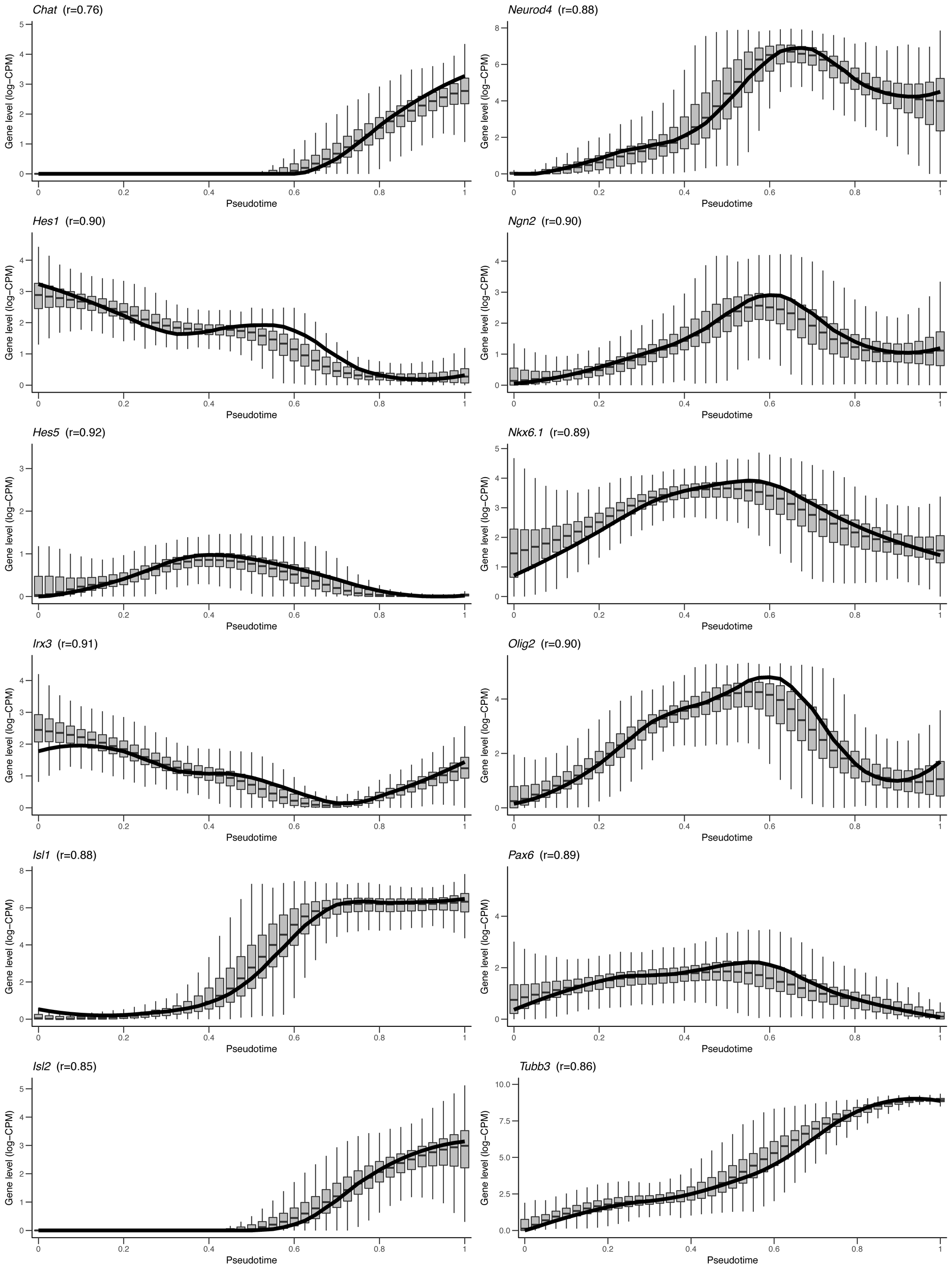

Supplement: S4 Fig — Solid lines indicate original gene levels shown in Figs 2E, 2F and S3E and S3F. At each pseudo–time point, box plots indicate median, first, and third quartiles of the gene level distribution obtained from 1,000 bootstrapped datasets. Whiskers indicate the largest and smallest values no further than 1.5 times the interquartile range, taken from the hinge. The associated correlation coefficient, r, is the average of the Spearman correlation coefficients over all pairs of boostrap replicates. (TIF) [file pbio.2003127.s004.tif]

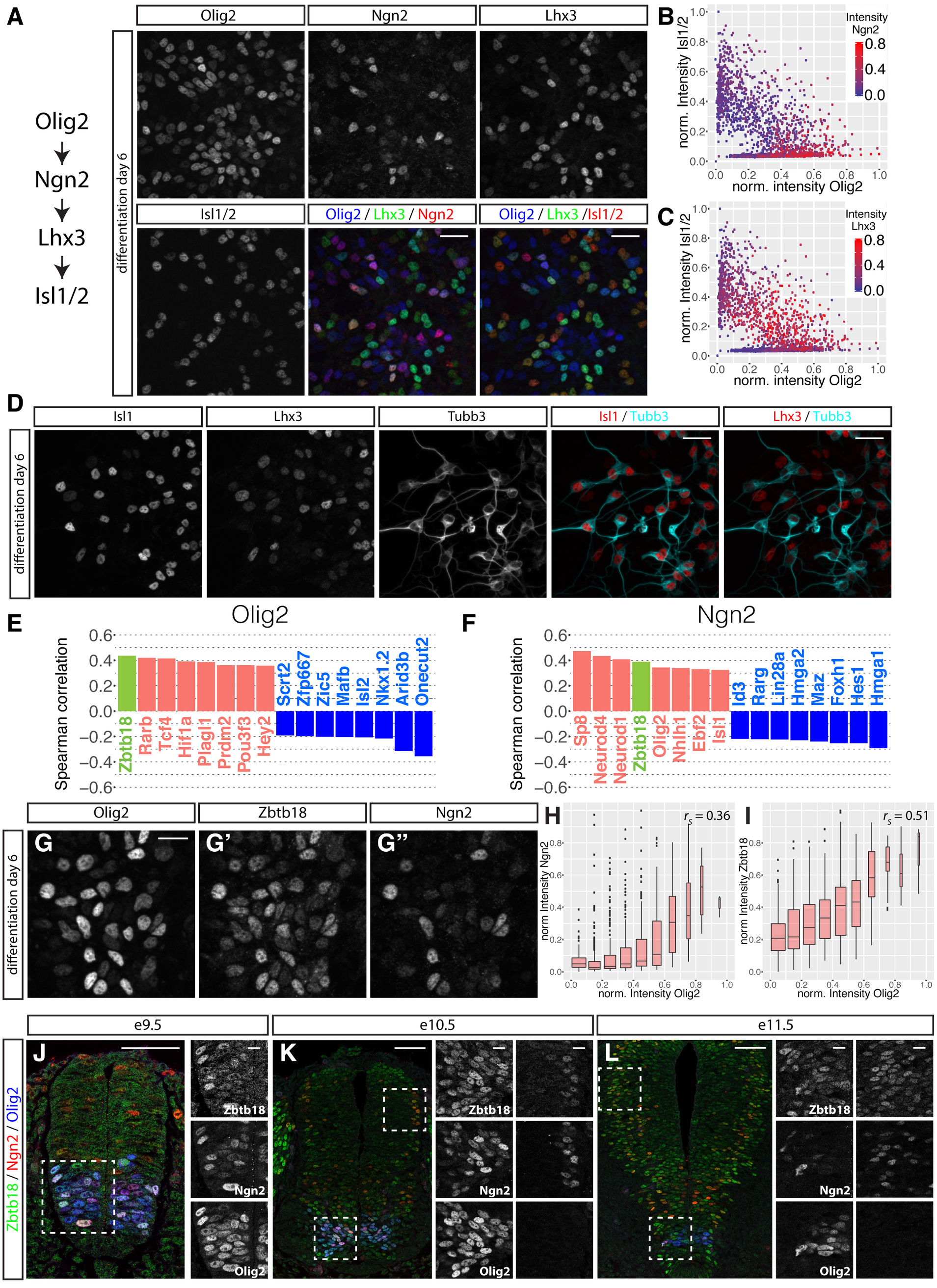

Supplement: S5 Fig — (A) Sequential expression of Olig2, Ngn2, Lhx3, and Isl1 during MN differentiation, revealed by immunofluorescent staining for these markers at day 6 of differentiation. (B,C) Quantification of levels of Olig2, Isl1, Ngn2 (color code in B), and Lhx3 (color code in C) reveals a clear differentiation path from Olig2HIGH cells to MNs and sequential induction of Ngn2 and Lhx3 during this process (n = 2,236 nuclei). Underlying data are provided in S1 Data. (D) Staining for Isl1, Lhx3, and Tubb3 reveals high levels of Tubb3 expression in Isl1-positive but not Lhx3-positive MNs at day 6 of differentiation. This is consistent with the earlier MN stage of Lhx3 MNs. (E, F) Most positive and negative Spearman-correlated transcription factors for Olig2 (E) and Ngn2 (F) reveal Zbtb18 (green in E–G) as a novel gene involved in MN formation. Underlying data are provided in S1 Data. (G–G″) Immunofluorescent staining for Olig2 (G), Zbtb18 (G′), and Ngn2 (G″) at day 6 of differentiation. (H, I) Quantification of levels of Olig2, Ngn2 (H), and Zbtb18 (I) in individual nuclei reveals a good correlation between these markers (n = 1,431 nuclei). Underlying data are provided in S1 Data. (J–L) Analysis of Olig2, Ngn2, and Zbtb18 expression in neural tubes at e9.5 (J), e10.5 (K), and e11.5 (L). Note that Ngn2 and Zbtb18 are expressed in cells with high levels of Olig2 at e9.5 and e10.5, but not at e11.5 (left insets in K–M). In addition, Zbtb18 and Ngn2 are coexpressed in nuclei at the edge of the progenitor domain in dorsal areas of the neural tube at e10.5 (L) and e11.5 (M) (right insets). Scale bars = 25 μm in (A,D), 10 μm in (G) and insets in J–L, 50 μm in J–L. e, embryonic day; MN, motor neuron; Tubb3, neuronal class III beta-tubulin. (TIF) [file pbio.2003127.s005.tif]

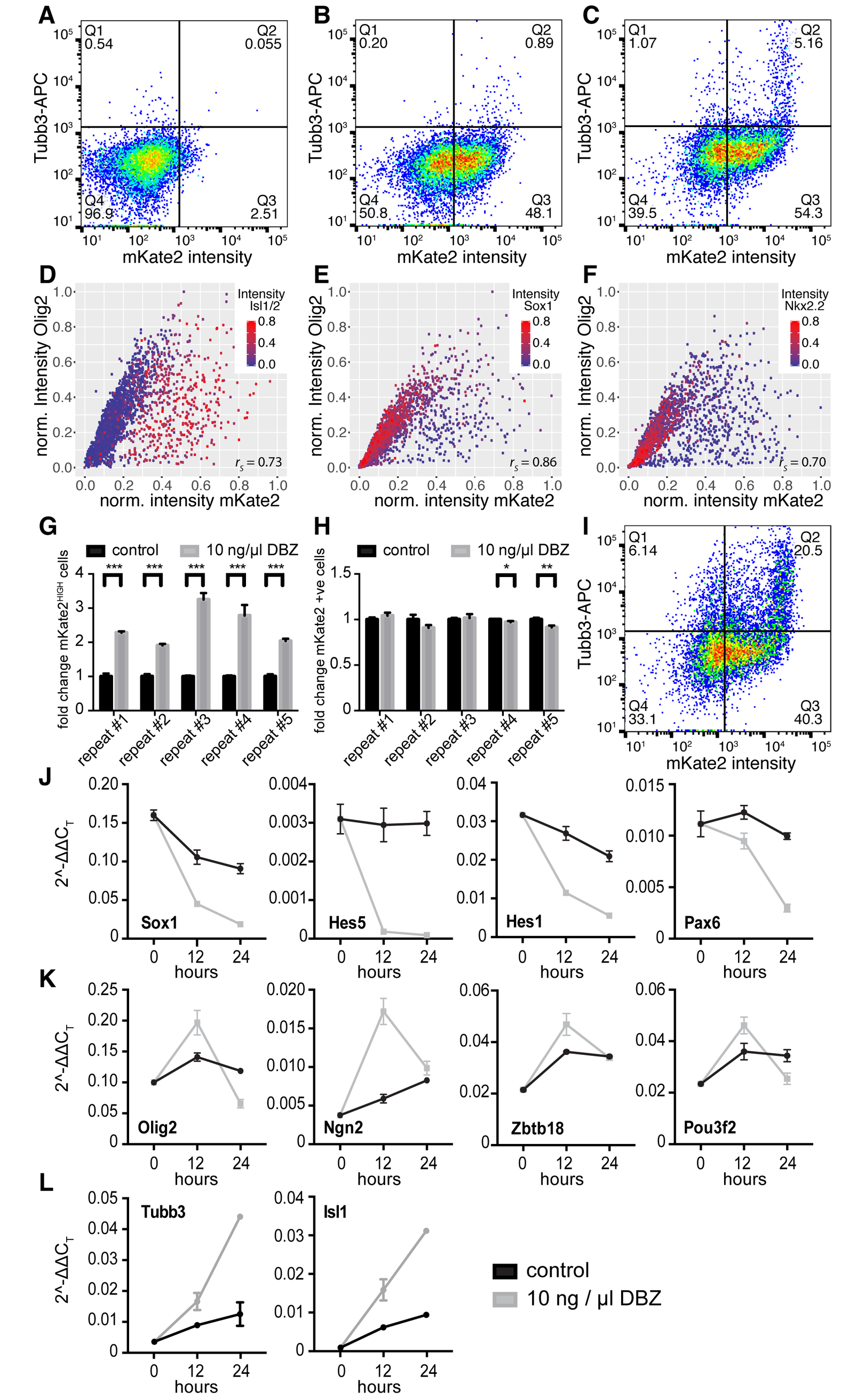

Supplement: S6 Fig — Characterization of the Olig2-mKate2 reporter cell line by flow cytometry and upon Notch inhibition (A–C) Quantification of mKate2 and Tubb3 fluorescence intensity by flow cytometry at day 4 to day 6 of differentiation. Note that high levels of Tubb3 are predominantly detected in mKate2HIGH cells at day 6 (C). (D–F) Correlation between Olig2 and mKate2 levels in individual nuclei quantified from images in Fig 4C–4F. Plots are color coded for levels of Isl1/2 (D), Sox1 (E), and Nkx2.2 (F). (G) Quantification of the fold change in mKate2HIGH cells (see Fig 4L) upon 24 hours Notch inhibition for five experimental repeats by flow cytometry. Each repeat consists of the measurement of three independent dishes for control and Notch inhibition from the same differentiation. Underlying data are provided in S1 Data. *** p < 0.001, unpaired t test. (H) Fold change of mKate2-positive cells (see Fig 4L) upon Notch inhibition (grey) relative to untreated control differentiations (black). Notch inhibition does not cause an overall change in the number of mKate2 positive cells. Underlying data are provided in S1 Data. * p < 0.05; ** p < 0.01, unpaired t test. (I) Quantification of mKate2 and Tubb3 fluorescence intensity by flow cytometry upon 24 hours Notch inhibition. Note that most mKate2HIGH cells differentiated into MNs (compare to S6C Fig). (J–L) RT-qPCR quantification of expression levels of progenitor markers Sox1, Hes5, Hes1, and Pax6 (J); neurogenesis markers Olig2, Ngn2, Zbtb18, and Pou3f2 (K); and MN markers Tubb3 and Isl1 (L) after 0, 12, and 24 hours of Notch inhibition (grey) and in untreated controls (black). Note that Olig2 expression increases in contrast to other progenitor markers after 12 hours of Notch inhibition. Underlying data are provided in S1 Data. MN, motor neuron; RT-qPCR, real time-quantitative polymerase chain reaction; Tubb3, neuronal class III beta-tubulin. (TIF) [file pbio.2003127.s006.tif]

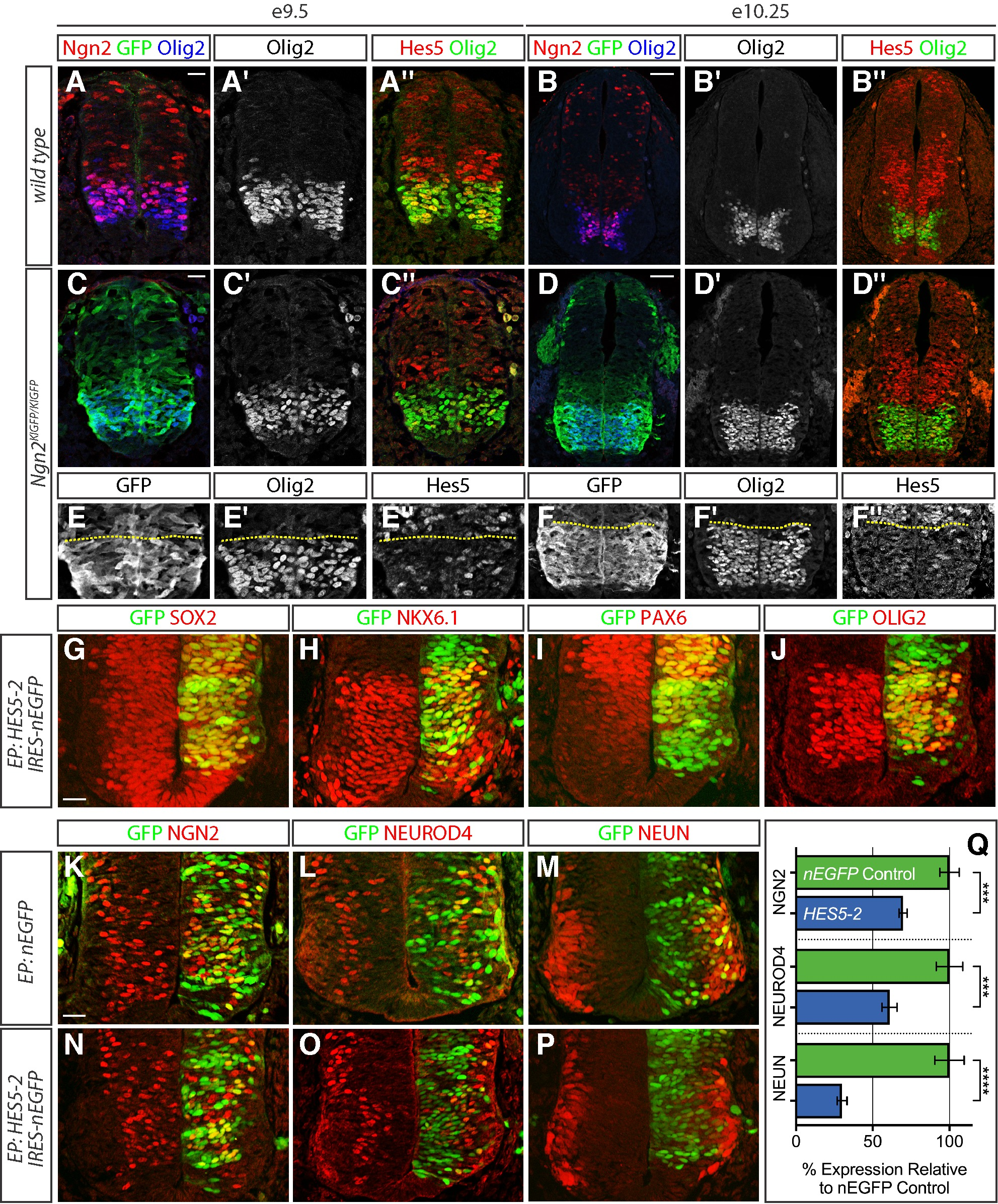

Supplement: S7 Fig — (A–D″) Staining of wild-type (A–B″) and Ngn2KIGFP mutant (C–D″) embryonic spinal cords for Olig2, Ngn2, GFP, and Hes5 at e9.5 (A,C) and e10.25 (B,D). (E–F″) GFP expression (E,F) is still increased and Hes5 expression (E″,F″) is still reduced in the pMN domain in Ngn2KIGFP mutant spinal cords (same sections as C–D″). The yellow dotted line indicates the dorsal boundary of the pMN domain. (G–J) Ectopic expression of cHES5-2 does not affect levels of the progenitor markers SOX2 (G), NKX6.1 (H), PAX6 (I), and OLIG2 (J). (K-P) Ectopic expression of cHES5-2 (N–P) leads to a reduction of NGN2 (K,N), NEUROD4 (L,O), and NEUN (M,P). (K–M) These show control electroporations with a nuclear EGFP (nEGFP) expression construct. (Q) Quantification of the effect of ectopic cHES5-2 expression on expression levels of NGN2, NEUROD4, and NEUN relative to nEGFP controls. Underlying data are provided in S1 Data. **** p < 0.0001; *** p < 0.0005, Mann-Whitney test; Scale bars = 20 μm (A–A″,C–C″,G–P), 50 μm (B–B″,D–D″). cHES5-2, chick HES5-2 gene; e, embryonic day; EGFP, enhanced green fluorescent protein; GFP, green fluorescent protein; MN, motor neuron; nEGFP, nuclear EGFP; pMN, MN progenitor. (TIF) [file pbio.2003127.s007.tif]

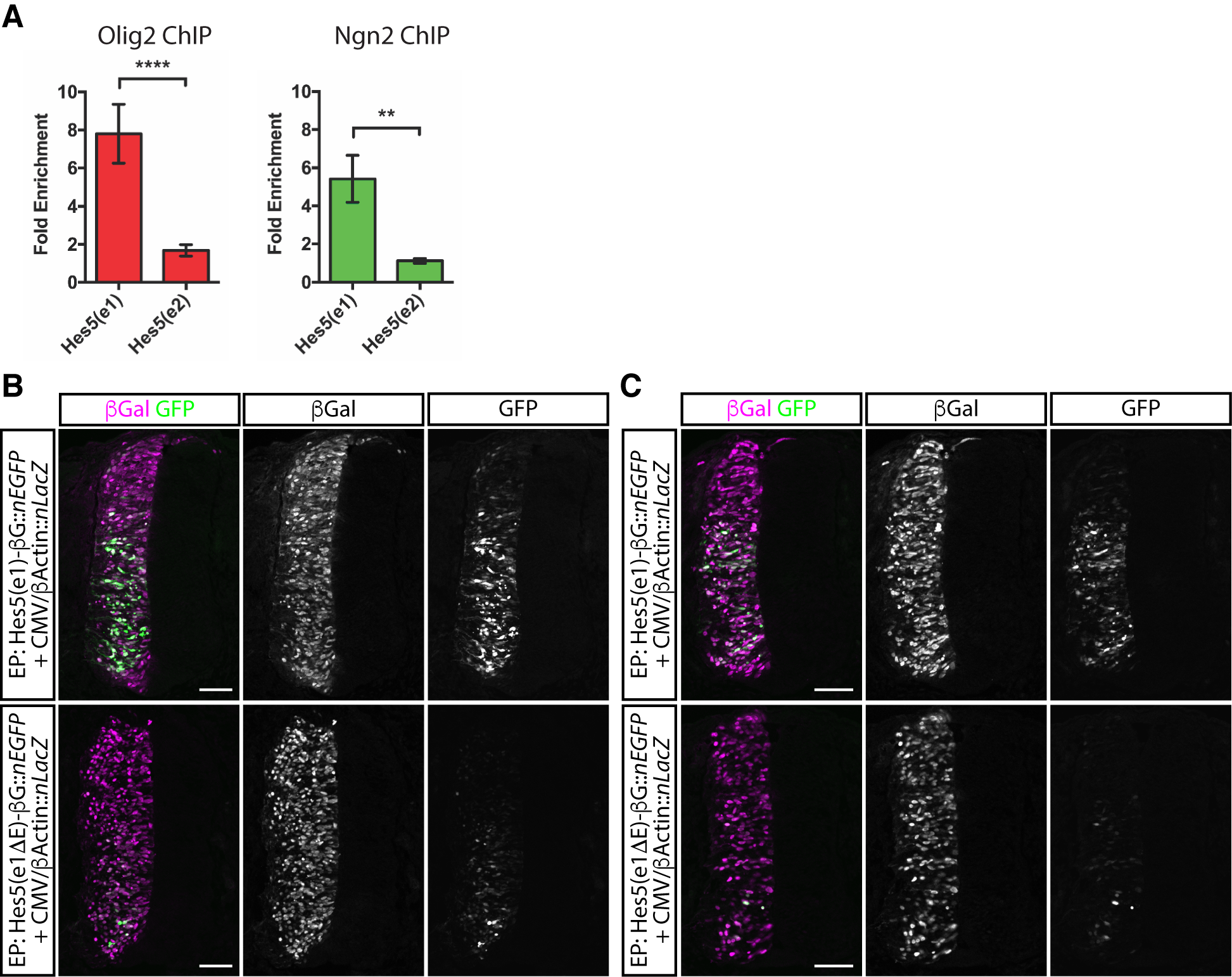

Supplement: S8 Fig — (A) Both Olig2 and Ngn2 antibodies precipitate the Hes5(e1) genomic element from ESC-derived MN progenitors, but not Hes5(e2), an unrelated genomic element 3′ to the Hes5 coding exons that also contains an E-box. Fold enrichment relative to normal rabbit sera or purified IgG is displayed. **** p < 0.0001; ** p < 0.01, Mann-Whitney test. Underlying data may be found in S1 Data. (B,C) Comparison between Hes5(e1)-βG::nEGFP (top row) and Hes5(e1ΔE)-βG::nEGFP (bottom row) reporter activities. Sections were imaged using identical settings for each pair. The overall activity of the Hes5(e1ΔE)-βG::nEGFP reporter is lower than that of the Hes5(e1)-βG::nEGFP reporter. Scale bars = 50 μm. E-box, bHLH protein binding site; ESC, embryonic stem cell. (TIF) [file pbio.2003127.s008.tif]
