# Supplementary material for: Olig2 and Hes regulatory dynamics during motor neuron differentiation revealed by single cell transcriptomics
Source: PLoS Biol. 2018 Feb 1;16(2):e2003127. doi: 10.1371/journal.pbio.2003127 (PMC5811045; doi:10.1371/journal.pbio.2003127)
Supplement: S1 Text — (DOCX) [file pbio.2003127.s012.docx]

**S1 TEXT**

**RNA sequences alignment and pre-processing**

Sequences were aligned to the Ensemble mouse genome GRCm38 using Tophat2 [1] and counted with HTSeq-count. Cell debris and doublets were removed from the data by inspecting miscroscope images of the microfluidic chips. Low-quality libraries were excluded from the 236 sequenced single-cell transcriptomes if their transcript abundance was less than 10^6^ reads and the number of expressed genes was less than one thousand. The 202 retained libraries (25 cells from day 4, 68 cells from day 5 and 109 cells from day 6) were normalized to read counts per million (CPM). Genes with counts in less than 3 cells or annotated as pseudogenes were excluded from the analysis.

**Cell state identification**

To identify the cell states in the dataset, we applied a two-stage strategy aimed at selecting the gene modules demonstrating relevant and concerted patterns of expression. First, we took a data-driven approach to characterize the different modules of interacting genes. From the initial set of 13196 expressed genes, we selected the 2287 genes that showed Spearman correlation (r > 0.4) with at least two other genes. The correlated genes were grouped into 127 gene modules by performing a hierarchical clustering using the Euclidean distance of the z-scored log-transformed gene levels and Ward's agglomeration criterion [2]. The number of modules was selected by determining the “elbow" position in the curve representing the total within-module gene level variation per number of modules. Gene modules were removed according to two criteria: insufficient number of cells expressing the comprised genes and inconsistent gene pattern in these cells. Both criteria were assessed by binarizing gene expression levels using an parameter-free adaptive thresholding method (R function binarize.array from the ArrayBin package). For each cell, we obtained an average expression level per module by averaging the z-scored log-transformed expression levels of all genes belonging to the module. Each of the 127 average expression level distributions were binarized independently. A cell was considered expressing a gene module if the associated Boolean value was true. Modules with fewer than four cells expressing it were excluded. The second criterion was designed to verify that cells expressing a gene module were showing consistently high levels over most of the genes composing the module. We binarized the z-scored log-transformed expression levels of all genes independently. Then, for each module, we calculated the ratio of Boolean values in cells expressing the module (as defined above). We excluded modules where less than half of these Boolean values were true. Twenty-two modules comprising 1064 genes were retained.

Second, functional annotation of the gene modules revealed the global and unbiased description of the biological processes represented in the dataset (see S1 Table and S2 Table showing the genes modules and their associated GO terms). In particular, two cell cycle-related gene modules were excluded (S2 Table): gene module 18 containing genes belong to cell cycle phases G2 and M, and primarily associated with the cell division GO term (GO:0051301); and gene module 20, containing G1 and S genes, and associated with the cell cycle GO term (GO:0007049). To focus on cell type characterization, we selected the 10 modules comprising the GO terms associated with embryonic development, i.e. nervous system development (GO:0007399), skeletal system development (GO:0001501), angiogenesis (GO:0001525), cell differentiation (GO:0030154).

**Cell population clustering**

In order to define the cell populations present in the dataset, we performed a hierarchical clustering (Ward's agglomeration criterion) of the Euclidean distances between cells using the z-scored log-transformed expression levels of the 545 genes included in the 10 selected modules (Fig 2A and S3A Fig). The 4 cell clusters containing vascular endothelial and mesodermal cells and the 5 associated gene modules were excluded from the subsequent analysis. 5 gene modules and 306 genes were retained.

**Single-cell state graph**

To investigate the dynamical changes of the transcriptional profile as cells differentiate, we developed a method to relate each cell to its closest neighbours in expression space. Unlike cluster analysis which aims to partition cells into groups with similar characteristics, hence breaking the continuity of cell state differentiation, we set out to generate graphs that connect individual cells without requiring the definition of groups. These can reveal the differentiation trajectories and intermediate states that link the clusters of similar cells (the “clustered" populations).

Using the log-transformed expression levels in the 306 genes space, we first calculated the Euclidean distance matrix between each cell and hence constructed a complete weighted graph of cell similarity D. In [3,4], a minimum spanning tree (MST) algorithm was used to extract the subset of cell-cell edges, which forms the backbone of differentiation branches. While MSTs ensure that all cells are connected, they are also sensitive to noise, making the local structure sensitive to small changes in the data [5]. To improve robustness to noise of MSTs, we constructed a consensus graph which combines multiple perturbed minimum spanning trees (pMSTs). Each pMST is obtained by calculating a MST from the cell dissimilarity matrix D with a certain ratio j of its elements set to a very large value (j=20%), hence forbidding the recruitment of the associated edges. Individual pMSTs are merged by summing their adjacency matrices into a matrix storing the occurrences of each edge. We then exclude rarely used edges by clustering the non-null edge occurrence distribution using the Fisher method [6] and removing all edges belonging to the first class. This leaves edges that are used repeatedly in multiple permutations and therefore represent good choices for inclusion in MST graphs. The perturb-and-merge algorithm works iteratively until convergence in the number of included edges. The graph visualization shown in Fig 2B,C and S3C Fig were obtained by projecting the graph into 2D where the positions of each cell (node) in the graph were initially random and then adjusted using an iterative force-based layout algorithm, ForceAtlas2 [7]. Gene expression patterns shown in Fig 2C and S3C Fig were smoothed by averaging each cell's log-transformed gene levels with its neighbors' log-transformed gene levels. We refer to these transformed levels as "log-smoothed" in the following.

**Pseudo-temporal ordering**

One of the advantages of generating a single-cell state graph is the possibility to infer a pseudo-temporal ordering of the gene expression by following the gene expression implied by the spanning tree. The strategy we used was to identify two terminal cell populations, early and late, and then find the K-shortest paths that connect each pair of early and late cells [8]. The early population was specified by selecting the 3 cells expressing a combination of highest Irx3 level and lowest Tubb3 level, and the late population by selecting the 3 cells expressing the highest Tubb3 level. A thousand k-shortest paths were generated for each of the 9 pairs of early and late cells. The resulting 9000 paths did not necessarily have the same length, 90.4% of them were formed by between 14 and 17 cells (shortest paths had 13 cells and longest 19 cells). In order to average gene expression along all paths, each of the 9000 paths was rescaled to the same length. Path rescaling was performed by replicating the cell IDs forming a path so that the total rescaled path length would match a constant value set to 41 pseudotime points (Fig 2D). As no path length was a factor of 41, some cell IDs were replicated either 2 or 3 times (13-cell-long paths being the exception with cell IDs replicated 3 or 4 times). For example, 16-cell-long paths had 7 cell IDs repeated 2 times, and 9 cell IDs repeated 3 times. To avoid the introduction of any bias in the repetitions, the choice of replicating a cell ID 2 or 3 times was random. The resulting 9000 equally-sized paths provided a list of 9000 cell IDs for each of the 41 pseudotime points. These lists allowed the calculation of various measurements along the pseudotime scale. In particular, Fig 2E,F and S3E,F Fig show the mean value of the 9000 log-smoothed gene levels for each of the 41 time points. All the pseudo-temporal dynamics were smoothed using a local polynomial regression fit (R function loess with span=0.5).

**Robustness of pseudo-temporal ordering**

In order to assess the robustness of our pseudo-temporal orderings, we performed a bootstrapping of the predicted 13 gene expression profiles shown in Fig 2 with 1000 replicates. Following standard bootstrapping procedure [9], the cells of each bootstrapped dataset were drawn randomly with replacement. Hence the bootstrapped datasets were composed on average of about 97 different cells while maintaining the original sample size with cells selected multiple times (the expected number of cells selected at least once in a boostrapped dataset is given by
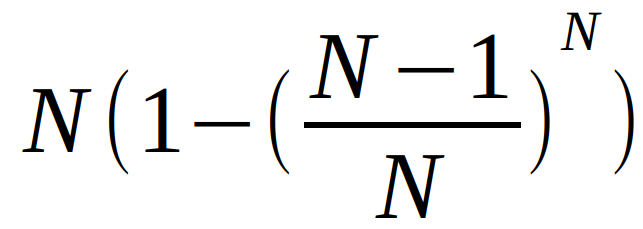
(with N=154 original cells). Following [10], we constructed a (1000 by 1000) "self-concordance" matrix for each gene, the elements of which are Spearman correlation of the expression profiles obtained between all pairs of replicates. Calculating the mean and standard deviation of these matrices reads: *Chat* (mean=0.76, sd=0.11), *Hes1* (mean=0.90, sd=0.05), *Hes5* (mean=0.92, sd=0.05), *Irx3* (mean=0.91, sd=0.07), *Isl1* (mean=0.88, sd=0.10), *Isl2* (mean=0.85, sd=0.13), *Lhx3* (mean=0.87, sd=0.12), *Neurod4* (mean=0.88, sd=0.11), *Neurog2* (mean=0.90, sd=0.08), *Nkx6.1* (mean=0.89, sd=0.08), *Olig2* (mean=0.90, sd=0.07), *Pax6* (mean=0.89, sd=0.06), *Tubb3* (mean=0.86, sd=0.09). The percentile confidence intervals for the gene expression profiles are shown in S4 Fig.

**Gene variation and dynamical states**

Quantification of the metastable states and transition phases were obtained by calculating the global gene variation along pseudotime. To do so, we identified the 2466 genes with higher dispersion, i.e. higher ratio of variance over mean as described in [11], and with an average expression level higher than 10 CPM to avoid taking into account low-level gene's variation. The absolute value of the first derivative of these genes was averaged to define the gene variation (Fig 2D,E,F and S3E,F Fig).

After applying differentiation-and-smoothing twice to gene variation (smoothing with local polynomial regression fit), we obtained a profile showing positive values for periods of higher gene variation and negative values for periods of lower gene variation, hence defining the dynamical states along pseudotime. This operation is equivalent to applying a low-pass Savitzky-Golay filter to the gene variation signal.

**References**

1. Kim D, Pertea G, Trapnell C, Pimentel H, Kelley R, Salzberg SL, et al. TopHat2: accurate alignment of transcriptomes in the presence of insertions, deletions and gene fusions. Genome Biol. BioMed Central; 2013;14: R36. doi:10.1186/gb-2013-14-4-r36

2. Ward JH. Hierarchical Grouping to Optimize an Objective Function. J Am Stat Assoc. 1963;58: 236–244. doi:10.1080/01621459.1963.10500845

3. Trapnell C, Cacchiarelli D, Grimsby J, Pokharel P, Li S, Morse M, et al. The dynamics and regulators of cell fate decisions are revealed by pseudotemporal ordering of single cells. Nat Biotechnol. Nature Publishing Group; 2014;32: 381–386. doi:10.1038/nbt.2859

4. Camp JG, Badsha F, Florio M, Kanton S, Gerber T, Wilsch-Bräuninger M, et al. Human cerebral organoids recapitulate gene expression programs of fetal neocortex development. Proc Natl Acad Sci U S A. National Academy of Sciences; 2015;112: 15672. doi:10.1073/pnas.1520760112

5. Zemel RS, Carreira-Perpiñán MÁ. Proximity Graphs for Clustering and Manifold Learning. In: Saul LK, Weiss Y, Bottou L, editors. Advances in Neural Information Processing Systems 17. MIT Press; 2005. pp. 225–232.

6. Fisher WD. On Grouping for Maximum Homogeneity. J Am Stat Assoc. 1958;53: 789–798.

7. Jacomy M, Venturini T, Heymann S, Bastian M, Diminescu D, Batagelj V, et al. ForceAtlas2, a Continuous Graph Layout Algorithm for Handy Network Visualization Designed for the Gephi Software. Muldoon MR, editor. PLoS One. Public Library of Science; 2014;9: e98679. doi:10.1371/journal.pone.0098679

8. Martins EV, Pascoal MB. A new implementation of Yen?s ranking loopless paths algorithm. Q J Belgian, French Ital Oper Res Soc. Springer-Verlag; 2003;1: 121–133. doi:10.1007/s10288-002-0010-2

9. Booth JG, Hall P, Wood ATA. Balanced Importance Resampling for the Bootstrap. Ann Stat. Institute of Mathematical Statistics; 1993;21: 286–298. doi:10.2307/3035591

10. Haghverdi L, Büttner M, Wolf FA, Buettner F, Theis FJ. Diffusion pseudotime robustly reconstructs lineage branching. Nat Methods. Nature Research; 2016;13: 845–848. doi:10.1038/nmeth.3971

11. Satija R, Farrell JA, Gennert D, Schier AF, Regev A. Spatial reconstruction of single-cell gene expression data. Nat Biotechnol. Nature Publishing Group, a division of Macmillan Publishers Limited. All Rights Reserved.; 2015;33: 495–502. doi:10.1038/nbt.3192
